# Metformin effects on cardiac parameters in non-diabetic Iraqi patients with heart failure and mid-range ejection fraction – a comparative two-arm parallel clinical study

**DOI:** 10.25122/jml-2023-0253

**Published:** 2023-09

**Authors:** Reeman Sabbar, Sinaa Abdul Amir Kadhim, Hayder Adnan Fawzi, Ali Flayih, Bassim Mohammad, Asma Swadi

**Affiliations:** 1 Department of Pharmacology, College of Medicine, University of Al-Qadisiyah, Al-Qadisiyah, Iraq; 2 Department of Pharmacy, Al-Mustafa University College, Baghdad, Iraq

**Keywords:** Heart failure, N-terminal proBNP, Echocardiography, Metformin, HF: Heart Failure, EF: Ejection Fraction, HFmrEF: Heart Failure with mid-range Ejection Fraction, LV: Left Ventricle, LVEDD: Left Ventricular End Diastolic Diameter, LVESD: Left Ventricular End Systolic Diameter, IVST: Interventricular Septal Thickness, NT-proBNP: N Terminal pro-brain natriuretic peptide, ST2: soluble suppression of tumorigenicity 2, HbA1C: Glycated Hemoglobin A1C, T2DM: Type 2 Diabetes mellitus, AMPK: Adenosine Monophosphate-Activated Protein Kinase, BMI: Body Mass Index, ARNI: Angiotensin receptor/Neprilysin inhibitor, ACEI: Angiotensin-converting enzyme (ACE) inhibitor

## Abstract

Heart failure (HF) remains a difficult challenge to the healthcare system, necessitating promoting interventions and multidrug management. Metformin, typically used to manage diabetes, has emerged as a promising intervention in the treatment of HF. This study aimed to assess the effect of adding metformin to the standard treatment of HF on cardiac parameters. This clinical study comprised 60 newly diagnosed HF patients randomly assigned to two groups: Group C received standard HF treatment, while Group M received standard HF treatment in addition to daily metformin (500 mg). After 3 months of treatment, group M showed a significantly higher ejection fraction (EF) compared to Group C (6.1% and 3.2%, respectively; p-value=0.023) and a reduction in the left ventricular end-diastolic pressure (LVEDD) (0.28, and 0.21 mm respectively; p-value=0.029). No significant differences were observed in the interventricular septal thickness (IVST) or left ventricular end-systolic pressure (LVESD). For cardiac markers, N-Terminal pro-BNP (NT-proBNP) showed the highest reduction in Group M compared to Group C (719.9 pg/ml and 271.9 pg/ml respectively; p-value=0.009). No significant changes were reported for soluble ST2. Metformin demonstrated cardiac protective effects by increasing EF and reducing NT-proBNP. Given its affordability and accessibility, metformin offers a valuable addition to the current HF treatment options. This positive effect may be attributed to mechanisms that enhance the impact of conventional HF treatments or vice versa.

## INTRODUCTION

Heart failure (HF) is a hemodynamic condition in which the heart fails to pump sufficient blood to the body or pumps blood at an inefficient pace due to excessive filling pressures [1, 2]. Symptoms of HF, such as dyspnea, tiredness, aberrant left ventricular (LV) and/or right ventricular filling pressure, and increased filling pressures contribute to the complexity of this clinical condition [3, 4]. Globally, HF affects an estimated 64.3 million individuals [5]. An estimated 1%-2% of the overall population is diagnosed with HF in developed countries [6].

The initial long-term management for patients with heart failure with reduced ejection fraction (HFrEF) includes the combined use of three types of agents, as tolerated: diuretics, a renin-angiotensin system blocker (angiotensin receptor/neprilysin inhibitor (ARNI), angiotensin-converting enzyme inhibitor (ACEI), or angiotensin receptor blocker (ARB)) or alternate therapy with isosorbide dinitrate-hydralazine, and a beta blocker, generally administered in the following order [7]. Evidence that initial therapies used in combination (beta-blocker and angiotensin system blocker (ARNI or ACEI)) prolong survival [7, 8] and reduce mortality compared to placebo was borderline significant for ARB therapy (used as an alternative to ARNI or ACEI) and for isosorbide dinitrate-hydralazine therapy (used as an alternative to an angiotensin system blocker) [9].

Metformin, a derivative of biguanide known as dimethyl biguanide, is considered one of the main drugs used to treat type 2 diabetes mellitus (T2DM) since its introduction in 1957. In addition, metformin has also demonstrated effects on cardiac cell function by altering cardiac metabolism and remodeling [10, 11]. Sixty to ninety percent of the heart's energy comes from fatty acid oxidation, with the rest coming mostly from glycolysis and the metabolism of lactate from the blood [12, 13]. As a result of decreased activity of the mitochondrial respiratory/electron transport chain, decreased utilization of fatty acids and glucose, and increased production of mitochondrial uncoupling proteins, the failing heart has an inadequate energy supply, resulting in decreased production of adenosine triphosphate (ATP) and phosphocreatine and decreased oxygen consumption [14, 15]. Metformin can improve cardiac function by affecting many metabolic pathways [16]. Metformin may increase mitochondrial oxidation of fatty acids by lowering the synthesis of the cardiac advanced glycated end product and decreasing cardiomyocyte apoptosis through activation of adenosine monophosphate-activated protein kinase (AMPK) [17]. The mechanisms of metformin in HF were the topic of two randomized clinical trials. Patients with insulin resistance and HFrEF were given metformin or a placebo for 4 months in one trial. The primary outcome (maximal VO2) was unaffected, while the efficiency of cardiac contractions increased [18]. Improved cardiac mechanical efficiency was also reflected in a substantial rise in the work metabolic index and a decrease in myocardial oxygen consumption compared to the placebo group. Patients with higher plasma metformin levels had greater changes in their work metabolic index than those whose metformin levels were lower [19]. The aim of the current work was to assess the effect of adding metformin to the standard treatment of HF on cardiac echocardiographic parameters (ventricular dimension-hypertrophy, septal thickness, and EF) and some cardiac biomarkers compared to the standard of care in heart failure with mid-range ejection fraction (HFmrEF).

## MATERIAL AND METHODS

### Study design and setting

**Figure 1:**
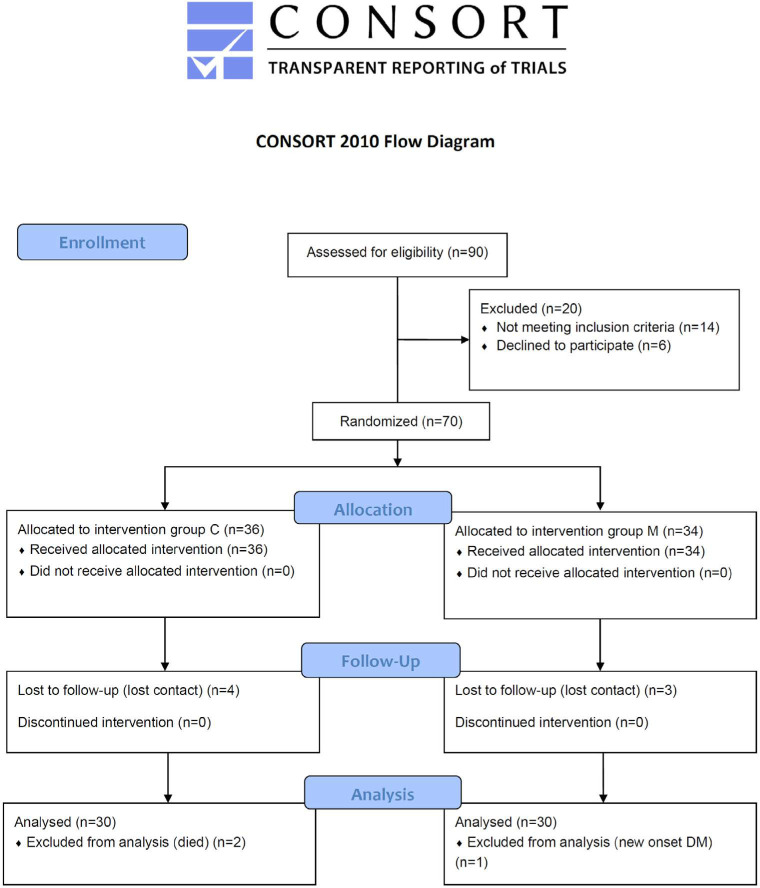
CONSORT flowchart of the study

Patients were recruited from the inpatient ward of Al-Diwaniyah Teaching Hospital in Al-Diwaniyah Province, Iraq, between September 1^st^, 2022, and March 1^st^, 2023. This prospective two-armed parallel-group clinical study involved 60 patients. We initially included 70 patients, but 10 dropped out during the follow-up (6 patients from group C and 4 from group M, as illustrated in Figure 1). The age range of participants was 50 to 70 years previously treated for new-onset HF (heart failure with mildly reduced EF with EF between 41 to 49%, HFmrEF) according to 2022 American Heart Association (AHA), American College of Cardiology (ACC), and Heart Failure Society of America (HFSA) guidelines [20]. Patients were divided into two groups: group C, treated with the standard treatment for HF, and group M, treated with the standard treatment for HF, in addition to Metformin 500 mg daily. The standard treatment for HF included Carvedilol (DILACARD^®^, MS Pharma, Jordan) 3.125 mg once daily, Bumetanide (Bumex^®^, Leo, Denmark) 1 mg once to twice daily, Sacubitril-valsartan (Savesto^®^, Gatz, Pakistan) 200 mg once daily, Spironolactone (Spironolactone^®^, accord, England) 25 mg twice daily, and group M was administered metformin (Glucophage^®^, Merck Serono, France) once daily with food given at evening.

All variables, including age, gender, body mass index (BMI), ejection fraction (EF), left ventricular end-diastolic diameter (LVEDD), left ventricular end-systolic diameter (LVESD), interventricular septal thickness (IVST), glycated hemoglobin (HbA1c) levels, N-terminal pro-brain natriuretic peptide (NT-proBNP) levels, and soluble suppression of tumorigenicity 2 (ST2) levels were collected at baseline and after three months, through echocardiography and biomarkers measurement.

### Inclusion and exclusion criteria

Inclusion criteria comprised non-diabetic patients newly diagnosed with HF stage II and III and HFmrEF. Exclusion criteria included individuals with renal impairment, age >70 years, intolerance to metformin, presence of cardiac or cerebral issues other than HF, systolic blood pressure equal to or greater than 180 mmHg, symptomatic hypotension, history of ketoacidosis, and any thyroid diseases.

### Randomization

Computer-based randomization was used. Patients were numbered consecutively and then randomized into two groups using the online software Research Randomizer.

### Sample size estimation

Sample size estimation was based on the following equation:


$$\text{minimum sample size (n)}=p\frac{(1-p)Z_{0.95}^{2}}{d^{2}}$$

Where n was the minimal sample size, p was the prevalence of HF, which, according to one study [6], was determined to be as high as 2% among the general population. The Z represents the Z-score at a 95 % confidence interval, which equals 1.96; d represents the marginal error, accepted at 0.05, according to one study [21]. The minimal sample size was estimated to be approximately 30 for each group.

### Analytic procedures

#### Sample preparation

A 10 ml venous blood sample was collected from each patient, and after collection, the blood was allowed to clot. The clot was later removed by centrifuging the sample at 2,000-3,000 rpm for 20 minutes. The resulting supernatant was divided into two portions. The first portion was used to assess serum lipid profile, urea, creatinine, and HbA1c, while the remaining supernatant was stored in a deep freeze (-80℃) until biomarkers analysis.

#### Human N-terminal Pro-Brain Natriuretic Peptide, NT-proBNP ELISA Kit (Sunlong, China)

For the detection of BNP sandwich, the ELISA method was utilized, which is based on antibody reaction to BNP antigen. The optical absorbance of the product was subsequently converted to concentration using the standard curve provided.

#### Human soluble ST2 ELISA kit (Sunlong, China)

For the detection of the ST2 sandwich – the ELISA method was utilized, which is based on the antibody reaction to the ST2 antigen. The optical absorbance of the product was subsequently converted to concentration using the standard curve provided.

#### Glycated Hemoglobin (HbA1c) (Linear, Spain)

The N-terminal fructosyl dipeptides of the HbA1c - chain were specifically measured by the hemoglobin A1c assay using an enzymatic technique, as mentioned previously [22].

### Echocardiography

All patients underwent echocardiographic assessment conducted by a specialist doctor using the Vinno G60 ultrasound system (serial number: 4011640003). The assessment included EF, LVEDD, LVESD, and IVST.

### Statistical analysis

All analyses were carried out using GraphPad Prism version 9.1 (GraphPad Prism (RRID: SCR_002798). To assess the adherence of variables to normality, the Anderson-Darling test was employed, and all variables were found to follow a normal distribution. Discrete variables are presented using numbers and percentages, with the chi-square test used to analyze these variables. Independent t-tests were used to assess the difference between both treatments, and paired t-test was used to assess changes between baseline and after 3 months in each group. A p-value was considered significant if it was less than 0.05, and all p-values were two-tailed.

## RESULTS

In this study, 60 patients were divided into 2 groups: Group C, receiving standard HF medications, and Group M, receiving standard HF treatment along with Metformin 500 mg daily. Each group consisted of 30 patients, with no significant differences in age, gender, or BMI between the two groups, which helped reduce selection bias.

**Figure 2:**
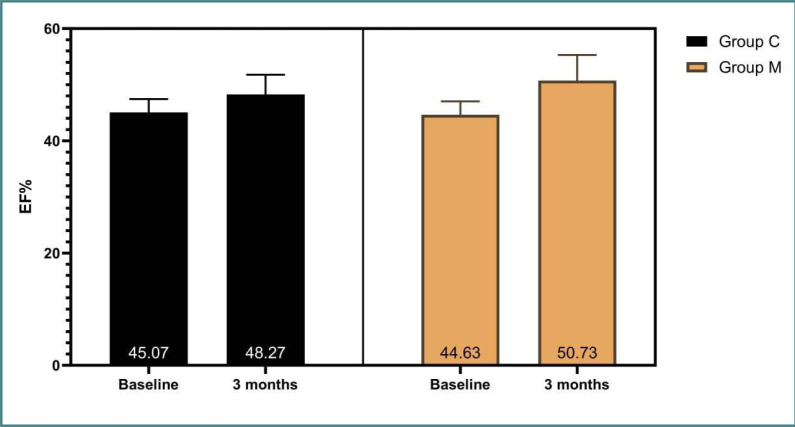
Comparison of EF assessment between the study groups (independent t-test)

There was a significant increase in EF in group M compared to group C (6.1% and 3.2% respectively, p-value=0.023) and a significant decrease in LVEDD pressure (0.28 and 0.21 mm respectively, p-value=0.029) between baseline assessment and the end of the study. There was no significant difference in LVESD, IVS thickness, or HbA1C between baseline assessment and the end of the study, as indicated in Table 1 and Figure 2.

**Table 1:** Assessment of demographic and echocardiographic characteristics

Variable	Group C	Group M	p-value
Number	30	30	-
Age (y), mean±SD	60.0±6.0	59.2±6.0	0.608^a^
Sex, n (%)			0.602^b^
Female	12 (40%)	14 (46.7%)	
Male	18 (60%)	16 (53.3%)	
BMI (kg/m^2^), mean±SD	26.6±2.9	26.1±2.9	0.520^a^
EF (%)			
Baseline	45.1±2.4	44.6±2.4	0.482^a^
After 3 months	48.3±3.5	50.7±4.6	0.023^a^ [S]
LVEDD (mm), mean±SD			
Baseline	6.69±0.38	6.55±0.36	0.141^a^
After 3 months	6.48±0.40	6.27±0.34	0.029^a^ [S]
LVESD (mm), mean±SD			
Baseline	4.85±0.36	4.86±0.39	0.946^a^
After 3 months	4.65±0.37	4.63±0.44	0.824^a^
IVS thickness (mm), mean±SD			
Baseline	0.88±0.19	0.86±0.17	0.614^a^
After 3 months	0.83±0.17	0.83±0.17	0.940^a^
HbA1c (%), mean±SD	4.97±0.32	4.99±0.32	0.749^a^

a Independent t-test, ^b^Chi-square test

S: Significant Difference, LVEDD: Left Ventricular End-Diastolic Diameter, LVESD: Left Ventricular End-systolic Diameter, IVS: Interventricular Septum, EF: Ejection Fraction, BMI: Body Mass Index, n: Number, HbA1c: Glycated Hemoglobin A1c, SD: Standard Deviation

The comparison was conducted at different times

**Figure 3:**
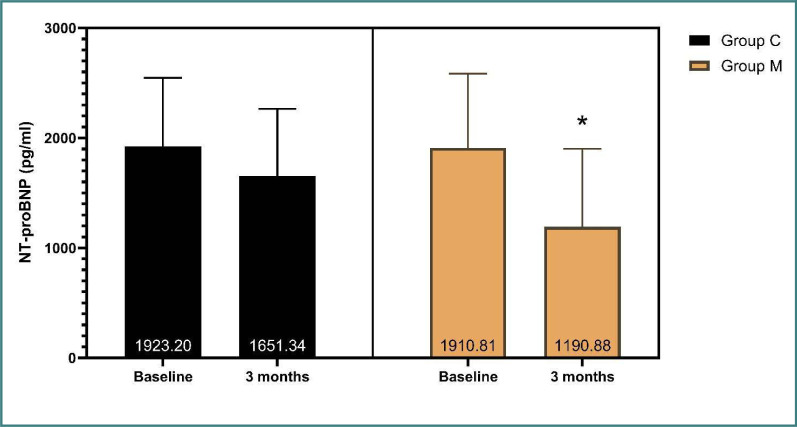
Assessment of NT-proBNP according to the study group (independent t-test)

A significant decrease was observed in the biomarker NT-proBNP after 3 months in group M compared to group C (719.9 pg/ml and 271.9 pg/ml, respectively, p-value=0.009). However, there was no significant reduction in ST2, as indicated in Table 2 and Figure 3.

**Table 2:** Assessment of cardiac biomarkers levels

Variable	Group C	Group M	p-value
Number	30	30	-
NT-proBNP (pg/ml), mean±SD			
Baseline	1,923.20±622.0	1,910.8±673.7	0.941
After 3 months	1,651.3±611.8	1,190.9±711.0	0.009 [S]
Mean reduction	271.9	719.9	
Soluble ST2 (ng/ml), mean±SD			
Baseline	30.00±2.00	30.05±2.80	0.937
After 3 months	25.95±1.59	24.99±2.26	0.061
Mean reduction	4.04	5.06	

Independent t-test

S: significant difference, SD: standard deviation

The comparison was made between groups at different times

## DISCUSSION

In the present study, all patients received standard treatment for HF according to recent guidelines, which included the administration of beta blockers, loop diuretics, an ACEI – neprilysin combination, and an aldosterone antagonist. The investigation explored the possible benefits of adding metformin in HFmrEF and its early benefits in HF in non-diabetic patients [23].

There is a lack of two-arm clinical studies focusing on patients with HFmrEF (LVEF, 41–49%), which underscores the importance of the current study, where metformin use in HFmrEF patients without diabetes appeared to improve HF in terms of EF and LVEDD. There was a significant increase in EF in group M compared to group C (6.1% and 3.2% respectively, p-value=0.023) and a significant decrease in LVEDD pressure (0.28, and 0.21 mm respectively; p-value=0.029) between baseline and the end of the study. Moreover, NT-proBNP showed a significant reduction in group M compared to group C (719.9 pg/ml and 271.9 pg/ml respectively, p-value=0.009). These findings suggest potential survival benefits for the patients, a factor not thoroughly examined in previous clinical studies.

Recently, there has been a change in the label of metformin use in HF, and the contraindication of use was removed. This change was based on a growing body of evidence highlighting the safety and advantages of metformin in individuals with diabetes and HF. This information was gathered from clinical observations and experimental investigations [24].

A meta-analysis of observational studies shows that metformin is safe for individuals with DM and HF, including those with decreased LVEF or chronic kidney failure. Conversely, no studies have shown that metformin increases the risk of lactic acidosis more than other hypoglycemic drugs [25].

A limited number of studies examined the relationship between metformin and non-diabetic HF. In a study that examined 6-month treatment with metformin in patients with metabolic syndrome, metformin improved EF compared to control (p-value <0.003) [26]. In another study that examined the effect of metformin in DM patients, metformin treatment reduced B-type natriuretic peptide (BNP) by 40% compared to the control group. BNP is an approved marker for diagnosis and therapeutic assessment of HF [27]. In a meta-analysis that involved 754 non-diabetic patients with LV hypertrophy, metformin treatment improved LVEF after 12 months of treatment [28].

In a study that examined 37 patients with HFrEF that received 3 months of treatment with metformin (in addition to standard of care) compared to the control group (which received standard of care), the authors found a 1% increase in EF compared to the control group [29]. These findings were consistent with another study by Wong and coworkers that examined non-diabetic HF, in which patients received 4 months of metformin and reported non-significant improvements (0.35±5.5) in EF compared to the control group [30]. In another study, metformin use was found to be a negative predictor of high BNP levels in patients with T2DM, indicating an inverse relationship between them [31].

According to the guidelines provided by the American Heart Association (AHA), American College of Cardiology (ACC), and Heart Failure Society of America (HFSA), assays for N-terminal pro-B-type natriuretic peptide (NT-proBNP) are commonly employed to determine the presence and severity of heart failure (HF). A reduction in NT-proBNP has been associated with better outcomes, underscoring the importance of NT-proBNP as a diagnostic and prognostic marker [20].

In the present study, both groups had a decrease in NT-proBNP levels after three months of follow-up, but metformin group M showed a significant reduction in NT-proBNP compared to the control group.

In a large study that examined diabetic patients with HF, the use of metformin was associated with a 16% reduction in HF events when compared to individuals not using metformin. The author concluded that metformin should be continued since the acute use of metformin showed the maximum benefit compared to non-use. Additionally, patients who discontinued metformin lost the benefits they had gained from its use [32].

Metformin was examined in several studies, including an exploratory analysis of the AVOCADO trial, which examined patients with T2DM at risk of developing HF, where metformin use was associated with low levels of NT-proBNP. Further analysis using a multivariate regression model found metformin to be independently associated with NT-proBNP [31]. Metformin medication may reduce the risk of HF by slowing LV remodeling. Given that NT-proBNP is an independent marker of developing HF, this might be very beneficial [31]. Experimental evidence from Sasaki *et al*. supports this idea by showing that metformin reduces cardiac remodeling and slows the development of HF in dogs while also increasing AMPK activation and nitric oxide (NO) generation [33]. Both AMPK activation [34-36] and NO generation [37] have been demonstrated to prevent cardiac remodeling in the pressure overload model and to reduce myocardial ischemia/reperfusion damage in the ischemic model in rats [38]. Using an experimental non-diabetic rat model of myocardial infarction (MI), Yin *et al*. showed that metformin decreased infarct size by 22%, leading to a 52% increase in LVEF compared to placebo [39].

The Wong *et al*. study showed a modest reduction in BNP compared to the control group (-20.2±78.7 pg/ml) [30]. In the Larsen *et al*. study, 4 months of treatment with metformin did not cause a significant change in BNP [29]. These findings raise the possibility that metformin's cardioprotective impact is unrelated to its ability to lower blood sugar.

In the present study, both groups had a significant decrease in soluble ST2 levels after three months of follow-up. Metformin did not show a statistical difference compared to the control group. No study examined the effect of metformin on ST2, and this study is the first to examine their effect on ST2.

ST2 is one of the promising new biomarkers for heart failure (HF). Numerous studies conducted in acute and chronic HF populations have shown that this biomarker holds predictive value when assessed individually and combined with other biomarkers [40].

One animal study that examined the effect of metformin on post-MI in rats found that metformin therapy reduced ST2 levels after 4-weeks of administration. This effect was associated with the downregulation of ST2 expression and the upregulation of IL-33, a protective marker in myocardial tissue that inhibits the phosphorylation of IκBα and the activation of NF-κB in the border tissue of cardiac muscles [41]. The lack of significant change in soluble ST2 levels observed in the current study following metformin treatment may be attributed to the relatively short duration of the study. It is possible that this marker may require a longer duration to achieve significant reduction.

This study has several limitations, including the short duration and the fact that no compensated morbidity and mortality outcomes were observed (because of the short duration). Finally, the open-label nature of the study might introduce bias in contrast to double-blinded trials.

## CONCLUSION

Metformin produced cardiac protective effects by increasing EF and reducing NT-proBNP. Its affordability and widespread availability make it a promising addition to existing HF therapies. This beneficial effect is possibly exerted by mechanisms related to a potentiation of the effect of standard treatment for HF or vice versa.

## Data Availability

Underlying data are available upon request from the corresponding author.

## References

[1] Pfeffer MA, Shah AM, Borlaug BA (2019). Heart Failure With Preserved Ejection Fraction In Perspective. Circ Res.

[2] Fawzi Hussein M (2012). Left Ventricular Remodeling Patterns in Chronic Heart Failure. Iraqi Postgrad Med J.

[3] Sharma K, Kass DA (2014). Heart failure with preserved ejection fraction: mechanisms, clinical features, and therapies. Circ Res.

[4] Reddy YN, Borlaug BA (2016). Heart Failure With Preserved Ejection Fraction. Curr Probl Cardiol.

[5] (2018). GBD, Global, regional, and national incidence, prevalence, and years lived with disability for 354 diseases and injuries for 195 countries and territories 1990-2017: a systematic analysis for the Global Burden of Disease Study 2017. Lancet.

[6] Groenewegen A, Rutten FH, Mosterd A, Hoes AW (2020). Epidemiology of heart failure. Eur J Heart Fail.

[7] Shah A, Gandhi D, Srivastava S, Shah KJ, Mansukhani R (2017). Heart Failure: A Class Review of Pharmacotherapy. P T.

[8] Theyab AA, Lee DS, Khachemoune A (2013). Angiotensin-converting enzyme inhibitor-induced angioedema. Cutis.

[9] Yancy CW, Jessup M, Bozkurt B, Butler J (2017). 2017 ACC/AHA/HFSA Focused Update of the 2013 ACCF/AHA Guideline for the Management of Heart Failure: A Report of the American College of Cardiology/American Heart Association Task Force on Clinical Practice Guidelines and the Heart Failure Society of America. Circulation.

[10] Han Y, Xie H, Liu Y, Gao P (2019). Effect of metformin on all-cause and cardiovascular mortality in patients with coronary artery diseases: a systematic review and an updated meta-analysis. Cardiovasc Diabetol.

[11] Ali ZS, Husain WM (2022). Metformin's hypolipidemic action in obese rats and its influence on leptin hormone levels. J Genet Environ Res Conserv.

[12] Stanley W (2001). Changes in cardiac metabolism: a critical step from stable angina to ischemic cardiomyopathy. Eur Heart J Suppl.

[13] Muslim AJ, Ali SA, Azal YH (2022). Cardiac energy and the level of tension in the aorta wall and its relationship to performance endurance Athletes have mild intellectual disability in youth football. Sci J Phys Educ.

[14] Brown DA, Perry JB, Allen ME, Sabbah HN (2017). Mitochondrial function as a therapeutic target in heart failure. Nat Rev Cardiol.

[15] Bertero E, Maack C (2018). Metabolic remodelling in heart failure. Nat Rev Cardiol.

[16] Dziubak A, Wójcicka G, Wojtak A, Bełtowski J (2018). Metabolic effects of metformin in the failing heart. Int J Mol Sci.

[17] Nesti L, Natali A (2017). Metformin effects on the heart and the cardiovascular system: A review of experimental and clinical data. Nutr Metab Cardiovasc Dis.

[18] Larsen AH, Jessen N, Nørrelund H, Tolbod LP (2020). A randomised, double-blind, placebo-controlled trial of metformin on myocardial efficiency in insulin-resistant chronic heart failure patients without diabetes. Eur J Heart Fail.

[19] Larsen AH, Wiggers H, Dollerup OL, Jespersen NR (2021). Metformin lowers body weight but fails to increase insulin sensitivity in chronic heart failure patients without diabetes: a randomized, double-blind, placebo-controlled study. Cardiovasc Drugs Ther.

[20] Heidenreich PA, Bozkurt B, Aguilar D, Allen LA (2022). 2022 AHA/ACC/HFSA Guideline for the Management of Heart Failure: A Report of the American College of Cardiology/American Heart Association Joint Committee on Clinical Practice Guidelines. Circulation.

[21] Daniel WW, Cross CL (2018). Biostatistics: a foundation for analysis in the health sciences. Wiley.

[22] Hirokawa K, Shimoji K, Kajiyama N (2005). An enzymatic method for the determination of hemoglobin A1C. Biotechnol Lett.

[23] Heidenreich PA, Bozkurt B, Aguilar D, Allen LA (2022). 2022 AHA/ACC/HFSA Guideline for the Management of Heart Failure: A Report of the American College of Cardiology/American Heart Association Joint Committee on Clinical Practice Guidelines. J Am Coll Cardiol.

[24] Wang J, Lu Y, Min X, Yuan T (2021). The Association Between Metformin Treatment and Outcomes in Type 2 Diabetes Mellitus Patients With Heart Failure With Preserved Ejection Fraction: A Retrospective Study. Front Cardiovasc Med.

[25] Eurich DT, Weir DL, Majumdar SR, Tsuyuki RT (2013). Comparative safety and effectiveness of metformin in patients with diabetes mellitus and heart failure: systematic review of observational studies involving 34,000 patients. Circ Heart Fail.

[26] Velázquez H, Meaney A, Galeana C, Zempoalteca JC (2016). Metformin enhances left ventricular function in patients with metabolic syndrome. Rev Mex Cardiol.

[27] Sokolova L, Pushkarev V, Cherviakova S, Vatseba T (2020). The effect of metformin treatment on the level of GLP-1, NT-proBNP and endothelin-1 in patients with type 2 diabetes mellitus. Международный эндокринологический журнал.

[28] Kamel AM, Sabry N, Farid S (2022). Effect of metformin on left ventricular mass and functional parameters in non-diabetic patients: a meta-analysis of randomized clinical trials. BMC Cardiovasc Disord.

[29] Larsen AH, Jessen N, Nørrelund H, Tolbod LP (2020). A randomised, double-blind, placebo-controlled trial of metformin on myocardial efficiency in insulin-resistant chronic heart failure patients without diabetes. Eur J Heart Fail.

[30] Wong AK, Symon R, AlZadjali MA, Ang DS (2012). The effect of metformin on insulin resistance and exercise parameters in patients with heart failure. Eur J Heart Fail.

[31] Rosiak M, Postula M, Kaplon-Cieslicka A, Trzepla E (2013). Metformin treatment may be associated with decreased levels of NT-proBNP in patients with type 2 diabetes. Adv Med Sci.

[32] Weir DL, Abrahamowicz M, Beauchamp ME, Eurich DT (2018). Acute *vs* cumulative benefits of metformin use in patients with type 2 diabetes and heart failure. Diabetes Obes Metab.

[33] Sasaki H, Asanuma H, Fujita M, Takahama H (2009). Metformin prevents progression of heart failure in dogs: role of AMP-activated protein kinase. Circulation.

[34] Russell RR, Li J, Coven DL, Pypaert M (2004). AMP-activated protein kinase mediates ischemic glucose uptake and prevents postischemic cardiac dysfunction, apoptosis, and injury. J Clin Invest.

[35] Shibata R, Sato K, Pimentel DR, Takemura Y (2005). Adiponectin protects against myocardial ischemia-reperfusion injury through AMPK-and COX-2-dependent mechanisms. Nat Med.

[36] Calvert JW, Gundewar S, Jha S, Greer JJ (2008). Acute metformin therapy confers cardioprotection against myocardial infarction via AMPK-eNOS-mediated signaling. Diabetes.

[37] Shibata R, Ouchi N, Ito M, Kihara S (2004). Adiponectin-mediated modulation of hypertrophic signals in the heart. Nat Med.

[38] Liao Y, Takashima S, Maeda N, Ouchi N (2005). Exacerbation of heart failure in adiponectin-deficient mice due to impaired regulation of AMPK and glucose metabolism. Cardiovasc Res.

[39] Yin M, van der Horst IC, van Melle JP, Qian C (2011). Metformin improves cardiac function in a nondiabetic rat model of post-MI heart failure. Am J Physiol Heart Circ Physiol.

[40] Aimo A, Vergaro G, Passino C, Ripoli A (2017). Prognostic Value of Soluble Suppression of Tumorigenicity-2 in Chronic Heart Failure: A Meta-Analysis. JACC Heart Fail.

[41] Asensio-Lopez MC, Lax A, Fernandez del Palacio MJ, Sassi Y (2019). Yin-Yang 1 transcription factor modulates ST2 expression during adverse cardiac remodeling post-myocardial infarction. J Mol Cell Cardiol.

